# Reply: Familial ovarian screening: how to address abnormal TVU findings and its influence on the efficacy of screening?

**DOI:** 10.1038/sj.bjc.6603409

**Published:** 2006-10-17

**Authors:** A L M Oei, L F A G Massuger, J Bulten, M J L Ligtenberg, N Hoogerbrugge, J A de Hullu

**Affiliations:** 1 Department of Obstetrics and Gynaecology, Radboud University Nijmegen Medical Centre, PO Box 9101, 6500 HB, Nijmegen, The Netherlands; 2 Department of Pathology, Radboud University Nijmegen Medical Centre, PO Box 9101, 6500 HB Nijmegen, The Netherlands; 3 Department of Human Genetics, Radboud University Nijmegen Medical Centre, PO Box 9101, 6500 HB Nijmegen, The Netherlands; 4 Department of Medical Oncology, Radboud University Nijmegen Medical Centre, PO Box 9101, 6500 HB Nijmegen, The Netherlands

**Sir**,

We are honoured with the opportunity to reply on the comment of Jacobs *et al* and on the comment of Mourits *et al.*

Firstly, Jacobs *et al*, who are experts in the field of surveillance of the ovaries, conclude that there remains a need for screening in younger women, who want to preserve fertility or avoid premature menopause for the following reasons: (1) lack of reliable data on annual surveillance in high-risk women (Hogg and Friedlander, 2004), (2) the underpowerment of our study on surveillance in high-risk women (Oei *et al*, 2006) and (3) Hogg and Friedlander (2004) suggested that annual screening does not have adequate sensitivity to detect early stage disease.

Above-mentioned comments may be true. On the other hand, especially in young women the number of women needed to screen to identify one patient with ovarian cancer will be extremely high, owing to the relatively low risk to develop ovarian cancer under the age of 40 (Antoniou *et al*, 2005). Moreover, this will lead to a higher number of additional interventions (diagnostic laparoscopies) owing to false-positive abnormalities in premenopausal women. Additional studies, mentioned in our publication, were not capable to detect all ovarian cancers nor ovarian cancer at an early stage disease in this group of high-risk women (Meeuwissen *et al*, 2005; Stirling *et al*, 2005; Vasen *et al*, 2005). In the study, population of Jacobs *et al* approximately 40% is above the age of 50 years. In our study the majority of the patients aged 50 years or up, underwent bilateral salpingo-oophorectomy (BSO).

We do agree with Jacobs *et al* that further research on early detection methods for ovarian cancer in this group of women should be undertaken. Hopefully, new methods can broaden the range of effective possibilities in the management of these high-risk women. We look forward to the results of the UK Familial Ovarian Cancer Screening Study (UKFOCSS) and the Cancer Genetics Network (CGN) CA125 studies initiated in the UK.

Secondly, we agree with Mourits *et al* that the presence of monolocular cysts should have been part of the method section especially because the majority of abnormalities at TVU were caused by these kind of cysts. In future studies these monolocular cysts should be identified and registered as a separate category. Regarding the issue on loss of quality of life in women with false-positive TVU findings as addressed by Mourits *et al*, our study is not capable to answer this question. Although we agree that quality of life is an important issue, our study was not designed to address this point.

In conclusion, until the results of earlier mentioned studies are available, we consider BSO the only evidence-based risk-reducing method for women at high risk of ovarian cancer (Kauff *et al*, 2002). Moreover, BSO also reduces the risk of breast cancer in premenopausal *BRCA* mutation carriers (Kauff *et al*, 2002). Short-term use of hormone replacement therapy after BSO have already been proven to be safe (Rebbeck *et al*, 2005) whereas Madalinska *et al*, (2005) showed that after BSO women experience fewer breast and ovarian cancer worries, and have a more favourable cancer risk perception. We feel obliged to counsel high-risk women for BSO from age 35–40 by emphasising the advantages of BSO and the limitations of surveillance.
